# A Female Psoriatic Arthritis Patient Involving the TMJ

**DOI:** 10.1155/2021/6638638

**Published:** 2021-02-11

**Authors:** Giovanni Falisi, Roberto Gatto, Carlo Di Paolo, Alberto De Biase, Carlo Franceschini, Annalisa Monaco, Sofia Rastelli, Gianluca Botticelli

**Affiliations:** ^1^Department of Life Health and Environmental Sciences, University of L'Aquila, L'Aquila 67100, Italy; ^2^Università degli Studi di Roma La Sapienza, Rome 00161, Italy

## Abstract

Psoriatic arthritis (PsA) is an inflammatory chronic arthritis associated with psoriasis. Currently, data about gender differences in clinical manifestation and therapeutic outcomes of PsA are limited. Frequently, women manifest a peripheral disease while men have an axial localization. Moreover, women display higher disease activity and physical activity limitations, if compared to men. Although the involvement of the temporomandibular joint (TMJ) is quite rare, it can seriously impact the quality of life. The morpho-functional peculiarities of TMJ require a multidisciplinary approach to perform a correct diagnosis and a successful treatment. Here, we report a case of a woman affected by PsA involving TMJ treated by combining pharmacological therapy and an occlusal splint. The coordination between different specialties led to a complete remission of clinical symptoms and a regression of lesions.

## 1. Introduction

Psoriasis is a chronic inflammatory relapsing and remitting pathology interesting skin but also nails, hair scalp, mucosae, and joints (5% to 42%) [1–3]. The epidemiological studies in literature report around 1 to 3% of incidence with prevalence in Caucasian ethnicity [4]. About 25% of patients with psoriasis develop a rheumatological form, the PsA [5, 6].

The gender prevalence of PsA is still under debate: a higher ratio of female to male was observed by Love et al., who considered an Iceland population [7]. Nossent and Gran found instead a male prevalence of the PsA [8]. However, the growing attention to the influence of the gender on the pathophysiology and management of diseases [9] stimulated researchers to investigate about any difference between the two genders in PsA [10–12]. There are growing evidences that the phenotype of the disease is different between male and female but further studies are needed [10, 13]. Sex differences in PsA include a more frequent axial involvement in men [6] and a predominant peripheral arthritis with higher disability scores in women [10].

*Ovarian hormones and pain response*: a review of clinical and basic science studies [14].

Indeed, women present multiple articular lesions, a significant lowering of the functionality and higher fatigue in the daily movements [11, 12, 15]. An important difference between genders is the response to therapy [10].

In addition, literature shows how the average age of diagnosis of the female gender is lower than male, and the beginning of the therapy usually overlaps with the peak of the reproductive age [15–17]. Another age peak is the postmenopausal age range, indicating a link with the impact of the hormonal fluctuations [11].

Also, the fluctuations and the hormonal peaks occurring during specific therapies, as, for example, ovarian hyperstimulation in reproductive techniques (ART) protocols, significantly impact on the course and onset of psoriasis [18].

Indeed, ART protocols include the administrations of follicular-stimulating hormones and gonadotropin antagonist, determining an important hormonal fluctuations and changes in the homeostasis of the female body system [16, 17, 19–22].

The psoriasis symptomatology and the form affecting the joints have a higher negative impact on the emotional and psychological life of women than in men [15].

The most common variety of psoriasis manifestation affects the skin and its annexes, but secondary manifestations in the oral cavity and temporomandibular joint (TMJ) can represent a challenging diagnosis.

Temporomandibular disorders (TMDs) are more frequent in psoriatic patients than in the general population [23, 24].

TMJ, as all the other complex joints can be affected by a form of PsA, is with negative implications to the patients' quality of life [25].

Here, we report a case, according to the CAse REport (CARE) guidelines [26], of a manifestation of PsA in the TMJ in a female patient including the peculiar diagnosis and treatment needed for this manifestation.

## 2. Case Description

### 2.1. The Patient: Reason of Attention, Medical, and Pain History

A female Caucasian patient, age 65, came to the attention of the dental clinic of University of L'Aquila complaining about a pain referred to the right ear. After a thorough otolaryngological examination, the ear did not present any inflammatory or infectious disease and medical attention was focused on the TMJ. The pain history revealed that the patient suffered from occasional pain to the right ear during the last 18 months, which became more frequent and acute in the days preceding the visit.

The patient referred that the pain was more acute upon waking up in the morning; it improved during the day and worsened only during mastication of solid and chewy food.

**Figure 1:**
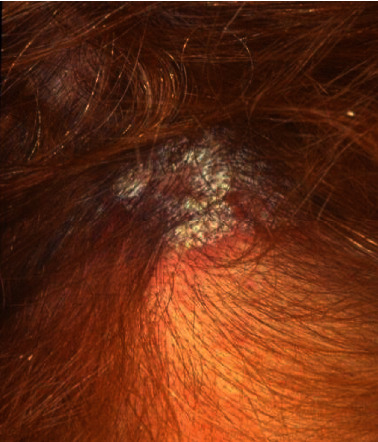
Psoriasis lesion on the patient's hair scalp.

The patient also referred that in the past 10 years, she noticed articular sounds coming from her temporomandibular joint. In addition, the patient revealed to suffer from a cutaneous form of plaque psoriasis on the hair scalp (Figure 1) and to be under control of the rheumatologist. The prescribed pharmacological medication for the psoriasis included no-steroidal anti-inflammatory drugs (NSAIDs).

Furthermore, the symptomatology began to appear two months after the suspension of the pharmacological medication when awaiting for the clinical analysis report for the new therapy for psoriasis.

### 2.2. Extra- and Intraoral Situation and TMJ Functional Examination

The extraoral examination did not reveal any facial asymmetry. The intraoral examination revealed a class 2 division malocclusion, according to the Angle classification [27]. The 4.6, 4.7 and 3.6, 3.7 elements were missing, determining the loss of the vertical posterior dimension.

**Figure 2:**
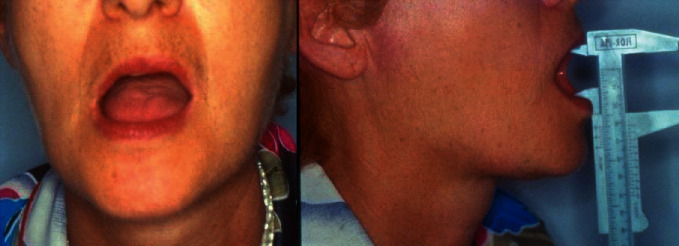
Limited open mouth measured by means of a mechanical calliper. Presence of laterodeviation towards the right side.

The TMJ functional exam highlighted a limited jaw opening with a right laterodeviation at the maximum mouth opening. The maximum jaw opening was measured by means of a mechanical caliber and was 32 mm (Figure 2).

Following is the clinical examination form of DC/TMD:

The patient referred pain in the right side of her face precisely at the level of the right TMJ without presenting any headache

Taking as a referral, the dental element 1.1, the patient presented 2 mm of overjet, 4 mm of over bite, and interincisive midline deviated to the right

The mandible was deviated to the right during opening and closing mandibular movements

Maximum opening with pain at 32 mm

Right lateral movement of 7 mm

Left lateral movement of 3 mm

Protrusion of 3 mm

Crepitus sounds at the right side of the TMJ during phases of opening, closing, and lateral movements

Pain during palpation at the right TMJ

The physical examination of the TMJ confirmed the presence of joint noises (crepitus) during jaw opening, protrusion, and lateral movements.

### 2.3. Pain Self-Perception, End-Feel Test, and Radiographic Evidence

The self-perception of the pain symptomatology was evaluated by means of the visual analog scale (VAS) [28]. At rest position, the VAS value was 6, while at the palpation, the VAS was 7.

The end-feel movement test was negative since no increased forced opening was appreciable.

**Figure 3:**
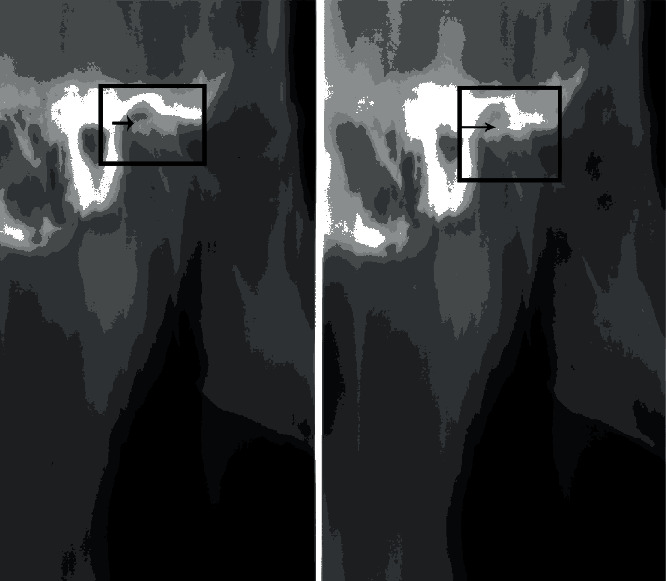
Stratigraphy of right TMJ. The arrows indicate the erosive changes on the surface of the condyle with interruption of cortical lining. When the articular eminence and the condyles are in contact, the joint space is narrow. Condyles and articular eminence appear both flattened.

The preliminary stratigraphy showed bone-remodeling signs on both condylar surfaces of the TMJ. The right temporomandibular joint has a greater remodeling than the left side, compatible with the symptoms reported by the patient. In addition, both the articular eminence of the zygomatic process and the upper surface of the condyle appeared flattened, resulting in a reduction of the intra-articular space (Figure 3).

### 2.4. Diagnosis and Treatment

The clinical and radiological information together with the medical history led to the diagnosis, according to the Diagnostic Criteria for Temporomandibular Disorders (DC/TMD) [29], of degenerative joint disease (ICD-9715.18) with right joint arthralgia (ICD-9 524.62).

The treatment, as recommended [29], was the causal therapy, and therefore, the patient was sent to the attention of the rheumatologist for the appropriate therapy (Brodalumab 210 mg, subcutaneous injection).

In addition, a stabilization appliance bite was prescribed as coadjuvant therapy to reduce the articular load on the TMJ.

Currently, there are several different occlusal splints available that have different clinical indications, making it hard for the clinician to choose the adequate one. At the moment, the scientific guidelines suggest more conservative treatments planned on the specific diagnosis.

The appliance chosen for this patient is an anterior repositioning stabilization splint.

The characteristics of this type of splint are a full upper teeth occlusal coverage with point-like contacts and the presence of an anterior pivot to allow mandibular protrusion.

**Figure 4:**
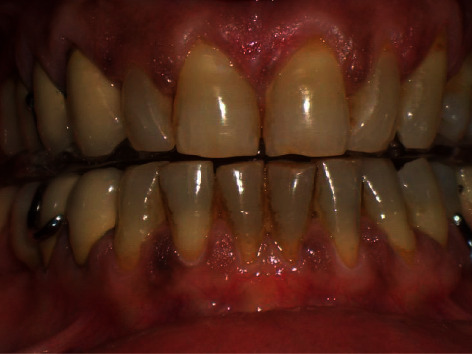
Reposition appliance DITRA.

**Figure 5:**
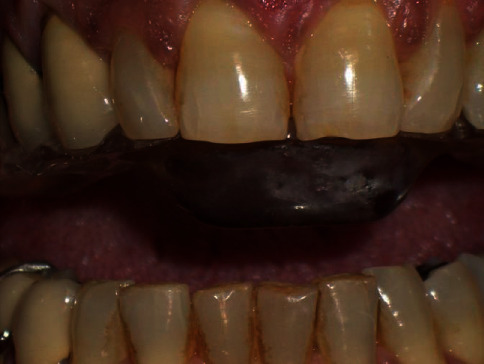
Pivot mandibular advancement.

The mandibular repositioning is three-dimensional because other than a sagittal movement, we will have an increase in the vertical and transversal dimension (DITRA) Figures 4 and 5 [30].

The time of application should not exceed one continuous hour per day and should be worn always during rest time either day or night.

The splint must be checked every 15 days to control the evolution of the patient's symptomatology.

### 2.5. Follow-Up

**Figure 6:**
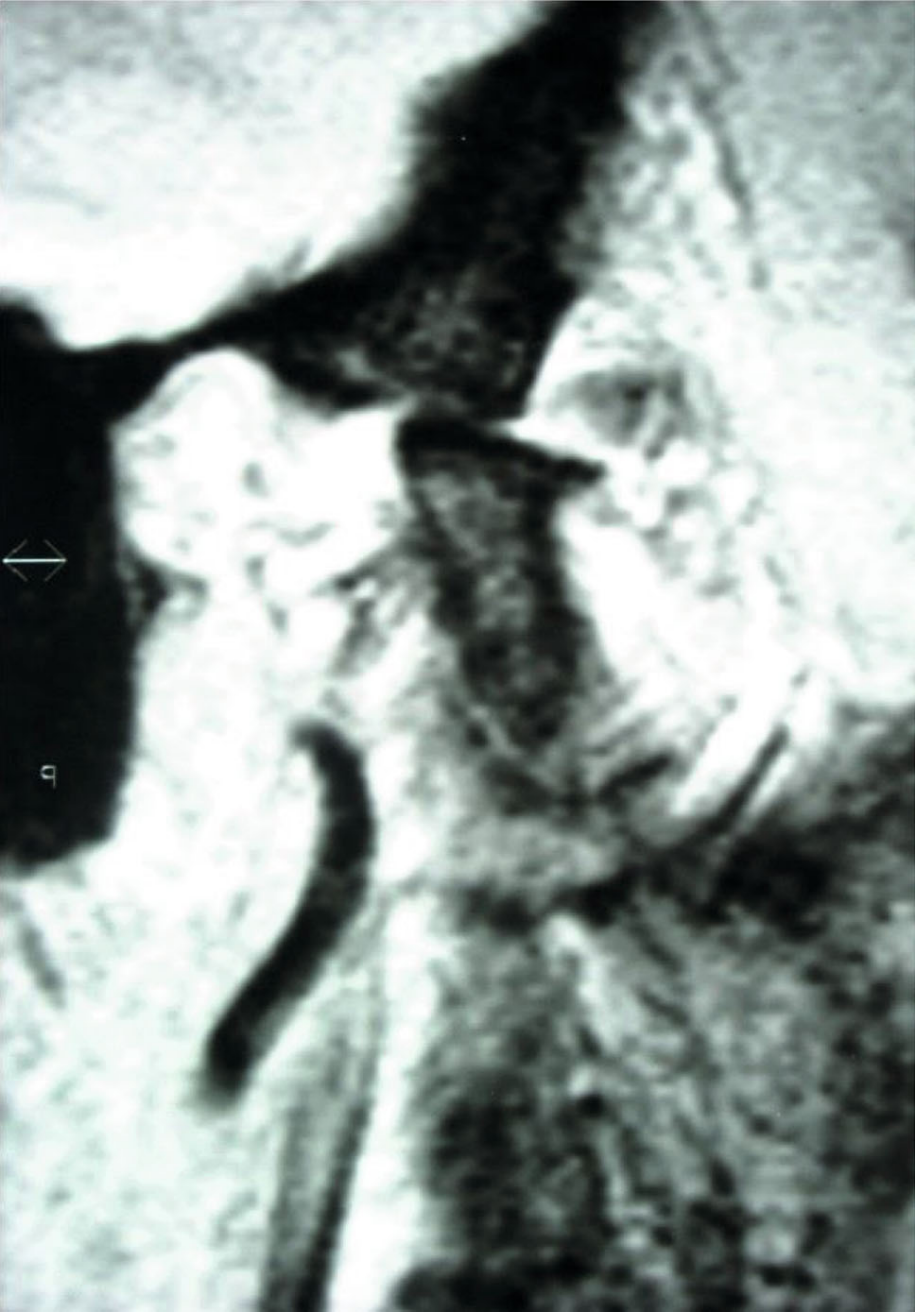
Representative MNR scan at 5-month follow-up. The erosive lesions on the condyle surfaces regressed, and interruption of cortical lining disappeared.

At the five-month follow-up, the patients referred a considerable lowering of the pain symptomatology. The VAS value at rest position was 2. In addition, the maximum mandibular opening improved to 40 mm. The mobility of the condyles also improved, as showed by the follow-up magnetic nuclear resonance (MNR) (Figure 6).

### 2.6. Ethics

The patient gave her written informed consent, in accordance with the Helsinki Declaration on the Ethical Principles for Medical Research Involving Human Subjects for the publication of her case report.

## 3. Discussion

Psoriasis can manifest in three forms: type I, type II, and PsA. Among these three forms, the latter increases the burden of the disease [11].

Cases of TMD due to PsA are reported in literature, but coherent statistical studies on its frequency are not available yet. Generally, the PsA forms affecting the TMJ are considered rare [1, 31–34].

However, an increased attention to the medical history of patients with rheumatologic disease is bringing up the number of diagnosed cases of PsA-TMD.

The female gender is generally more exposed to develop TMDs, and the disturbs affecting this joint significantly decrease the quality of life of the patients, due to the secondary symptomatology such as general orofacial pain, headache, myalgia at masticatory muscle level, and sleep disorders [35–37].

Christidis et al. reported the higher expression in women at masseter levels of the serotonin receptor, increasing the sensitivity of the female sex to the pain in the TMJ-masseter region [38].

This condition, in combination with a peculiar sensitivity to pain stimulation due to the sex hormonal peaks and changes [39], expose the female gender to a higher burden in case of manifestation of TMJ arthropathy.

PsA negatively affects the quality of life of women [23], and the development of this form in the TMJ, as rare as it can be, results in a worsening of the clinical conditions.

Indeed, the complexity of the PsA requires regular follow-ups and long-life treatments that might need adjustments over the time, generating frustration in the affected patients [15].

The diagnosis of PsA-TMD is based on a careful medical history assessment, on an accurate physical examination of the TMJ and the masticatory muscles and on the radiological evidences.

The clinical signs of a TMJ affected by PsA are represented by limited functionality, muscular pain, clicking or crepitation sounds, and altered opening derangement [31].

The radiological signs obtained using magnetic resonance or CT scans reveal an alteration of the morphology of the condyles and of the mandibular fossa, with signs of erosion, bone remodeling, and osteoarthritis [40].

The radiographic image of the examined patient showed a greater anatomical structural alteration of the right condyle. There is a direct proportionality between the structural modifications of the TMJ and the symptoms reported by the patient.

The therapy is causal [41] and therefore should be aimed at controlling the disease; for this reason, the rheumatologist should be alerted of the diagnosis to modulate the right pharmacological therapy to control the disease.

As reported by Wilson et al., the pharmacological plan should include the administration of NSAIDs and steroids [42]. In addition, coadjuvant therapies, such as physiotherapy, may help in the pain reduction and the functionality recovery [42]. Another therapy option for the treatment of PsA-TMD is surgery, which should be reserved to those cases where pharmacological therapy is not successful.

In the presented case, beyond the causal therapy, an occlusal appliance bite was prescribed and added as coadjuvant therapy.

The preferred therapy in TMDs consists in the use of stabilization splints as in the short term; it is the one that shows clear improvements in symptoms.

The repositioning splint used, since it is built in a slight protrusion position, causes a lowering of the activity of the masseters by reducing the vertical muscular load which could increase intra-articular pressure, with a consequent improvement in symptoms. In addition, the main function of the occlusal splint is to carry out a protective activity on the joint structures, because the nocturnal phase parafunctional muscular phenomena could occur leading to an increase in the symptomatological set.

Indeed, the TMJ is a peculiar joint, allowing not only the simple mouth opening and closure, but also contributing to other movements such as swallowing, chewing, sucking, breathing, phonatory acts, and facial mimicry [41, 43, 44]. Hence, the mechanical stress due to the physiological movements makes the TMJ a delicate and complex structure to be approached.

Intervening on occlusal interferences helped in the improvement of the TMJ functionality and confirmed how a patient affected by such complex disease can benefit from a therapeutic multidisciplinary approach.

## 4. Conclusions

Dental operators and the specialists of TMJ should be involved in a global view of the treatment of female psoriatic patients, and conversely, they should pay attention when facing this special category of patients.

The availability of targeted treatment for the systemic disease in association with an appropriate supportive therapy to the joint positively improves the quality of life of women affected by PsA on the TMJ.

## Data Availability

Data are available on request.
